# Multiple paradoxical embolisms revealing a patent foramen ovale in a patient with deep venous thrombosis: A case report

**DOI:** 10.1016/j.amsu.2021.102426

**Published:** 2021-05-28

**Authors:** Youssef Banana, Abdellah Rezziki, Oussama Kallel, Hammam Rasras, Zakariae Bazid, Noha El Ouafi, Omar El Mahi, Adnane Benzirar

**Affiliations:** a Department of Vascular Surgery, Mohammed VI University Hospital of Oujda, Mohammed First University of Oujda, Morocco; b Department of Cardiology, Mohammed VI University Hospital of Oujda, Mohammed First University of Oujda, Morocco; c Laboratory of Epidemiology, Clinical Research and Public Health, Faculty of Medicine and Pharmacy, Mohammed the First University of Oujda, Morocco

**Keywords:** Paradoxical embolism, Acute ischemia, Patent foramen ovale, Embolectomy, Case report

## Abstract

**Introduction:**

Paradoxical embolism is a rare medical phenomenon. Depending on the site of embolisation, it can cause different symptoms. Although rare, mesenteric ischemia can reveal paradoxical embolism, and the embolisation of two different sites is rarely described in the literature.

**Case presentation:**

We report the observation of a patient with a table associating an acute mesenteric ischemia and an acute ischemia of the upper limb; whose the etiological assessment revealed a deep venous thrombosis of the lower limbs complicated by pulmonary embolism.

**Clinical discussion:**

These paradoxical embolisms occurred through a patent foramen ovale. The diagnosis of the patent foramen ovale in this patient was revealed by transthoracic echocardiography, with bubble test. The patient benefited from an embolectomy of the superior mesenteric artery and an embolectomy using fogarty catheter by approching humeral artery at the elbow crease with good postoperative evolution. The patient was put on long-term anticoagulation with Acenocoumarol (because of low socio-economic level of our patient). We didn't recommended the closure of the PFO because of the small size of the shunt and especially because the patient refuses that procedure.

**Conclusion:**

Paradoxical embolism remains a pathology rarely mentioned by clinicians, although it can engage the functional and vital prognosis of the patient, hence the interest of a good cardiac evaluation in any patient with embolic ischemia.

## Highlights

• PFO is most often asymptomatic but it can cause manifestations with variable degrees of severity.
• If it is not earyl diagnosed, or negligated, it can be life-threatining
• Paradoxical embolism is a pathological condition that requires a multidiscplinary management.

## 1 Introduction

Paradoxical embolism was described for the first time by Connheim in 1877, and defined as the embolic entry of venous thrombosis into the systemic circulation through a right-to-left shunt [1]. Depending on the site of embolisation, paradoxical embolism can cause an ischemic stroke which is the most frequently described [2], a myocardial infarction [3], an acute mesenteric ischemia [4], a kidney infarction [5], or an acute ischemia of the limbs [6]. The diagnosis of paradoxical embolism requires a thrombus in the venous circulation, a communication between the right and the left heart chambers with a pressure gradient that should be responsible of the presence of an embolism in the systemic circulation [6]. The anatomical abnormality associated with paradoxical embolism represented in 70% of cases by inter-country communication linked to a patent foramen ovale (PFO) [7]. More rarely, it is a deficiency of the atrial septum or the ventricular septum, pulmonary arteriovenous malformation, anomaly of Ebstein or of a ductus arteriosus [8].

We report the case of a patient who presented with an acute mesenteric ischemia and an acute ischemia of the upper limb, whose the etiological assessment revealed a deep vein thrombosis of the lower limbs complicated by pulmonary embolism.

Our case report was written according to SCARE guidelines [9].

## 2 Presentation of case

**Fig. 1:**
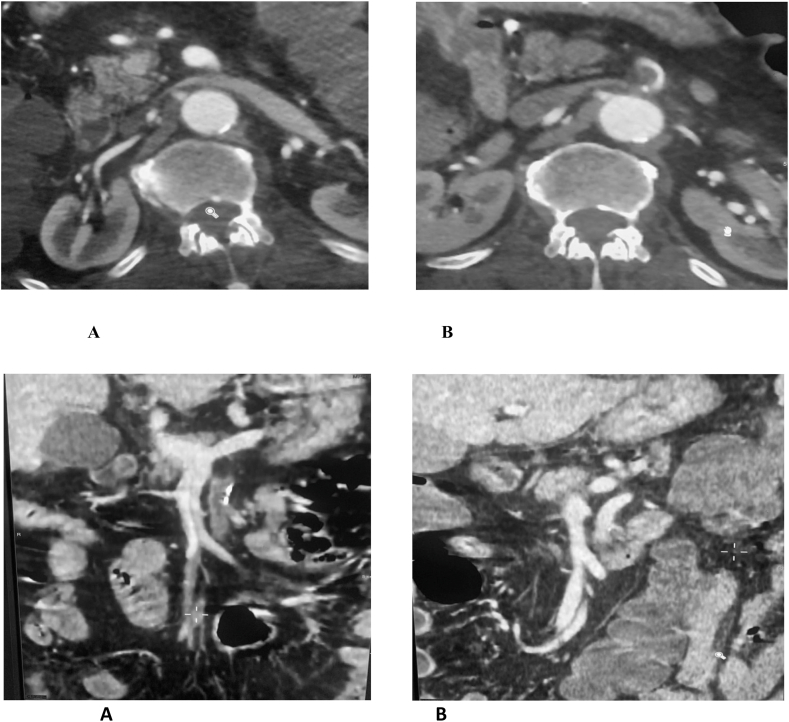
Transverse and coronal plane showing a thrombus at the origin of the superior mesenteric artery before A and after B embolectomy.

A 76-year-old man, with a past medical history of diabetes mellitus and high blood pressure, was admitted to the emergency room for an acute superior mesenteric ischemia confirmed by abdominal angiography CT scan (Fig. 1). On physical examination, the patient was afebrile, whith a blood pressure at 110/73 mmhg, heart beats at 112 beats per minute, left leg swelling with a sign of HOMANS +, without sign of shock. Blood analysis showed: white blood cells at 19810, C-reactive protein at 179 mg/L; hemoglobin at 12.9, serum creatinine at 8.50; urea at 0.40, with an arteriel gas test objectified a lactate level at 4.

Transthoracic echocardiography (TEE) showed: a left ventricle with good systolic function, a dilated right ventricular with conserved systolic function, systolic pulmonary artery pressure (SPAR) at 45 mmhg, with aneurysm of the atrial septum. A complementary by Thoracic CT angiography revealed a bilateral pulmonary embolism. Venous doppler of the lower limbs showed a deep venous thrombosis in the left inferior limb.

**Fig. 2:**
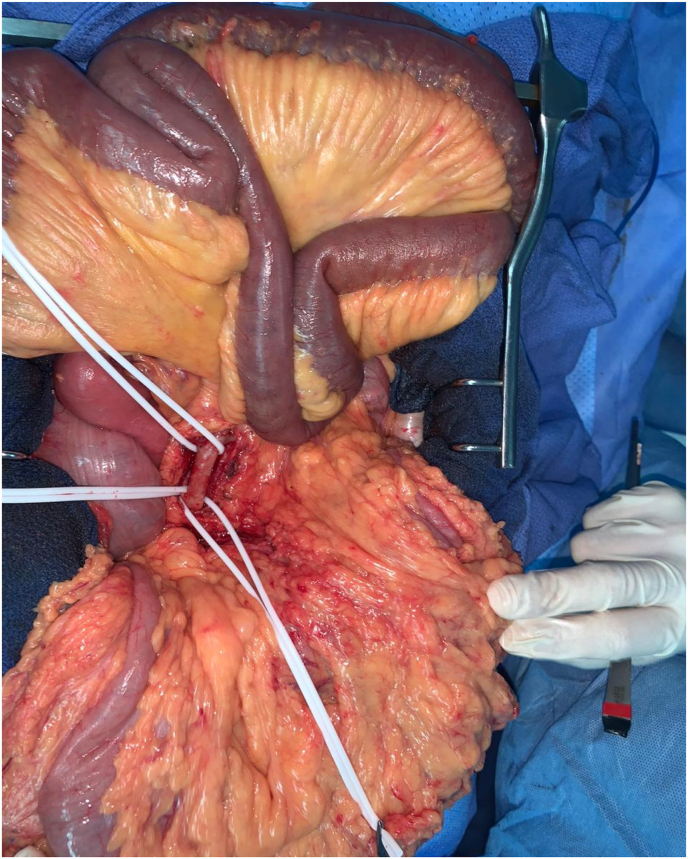
At the exploration partiel discoloration of the small intestine with control and placing on lakes of the superior mesenteric artery.

**Fig. 3:**
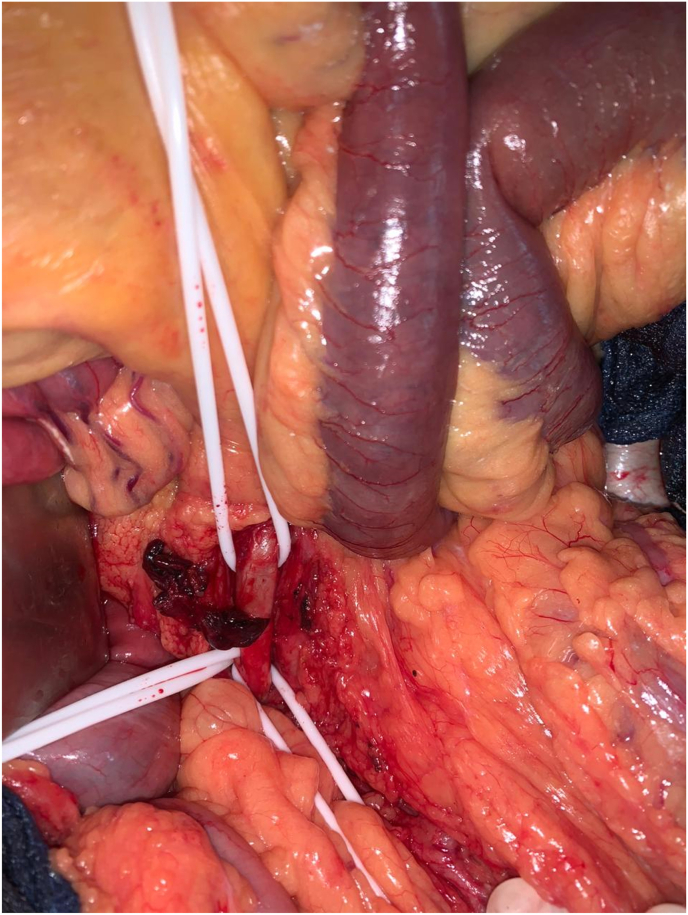
A fresh fibrin cruoric clot after thrombectomy with the fogarty catheter of the superior mesenteric artery.

**Fig. 4:**
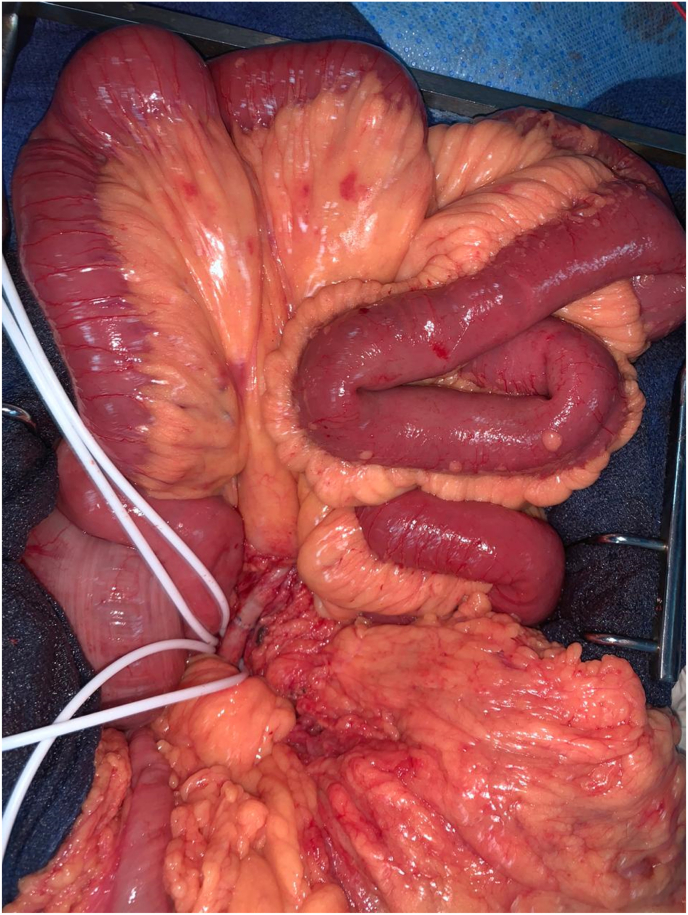
After suturing of the arteriotomy, visualization of a progressive recoloration of the small intestine.

According to the state of emergency, the patient has benefited an exploratory laparotomy. At the exploration: no intestinal necrosis, with partiel discoloration of the small intestine. (Fig. 2), then he benefited from an embolectomy of the superior mesenteric artery by median xyphopubien laparotomy by approaching the superior mesenteric artery in its intramesenteric segment with embolectomy of its branch colica media, this technique was realized by a FOGARTY 5F catheter (Fig. 3), with a progressive recoloration of the small intestine (Fig. 4). The post operative evolution was favourable, with resumption of intestinal transit the next day. At the postoperative third day, the patient presented an acute ischemia of the right upper limb, then he benifited an embolectomy using fogarty catheter (3F) by approching humeral artery at the elbow crease, with issue of a fresh fibrinocruoric clot, with a good post operative improvement. The surgical management of this case was performed by an experienced professor of vascular surgery with the aid of an assistant professor and 2 junior residents in the same speciality.

**Fig. 5:**
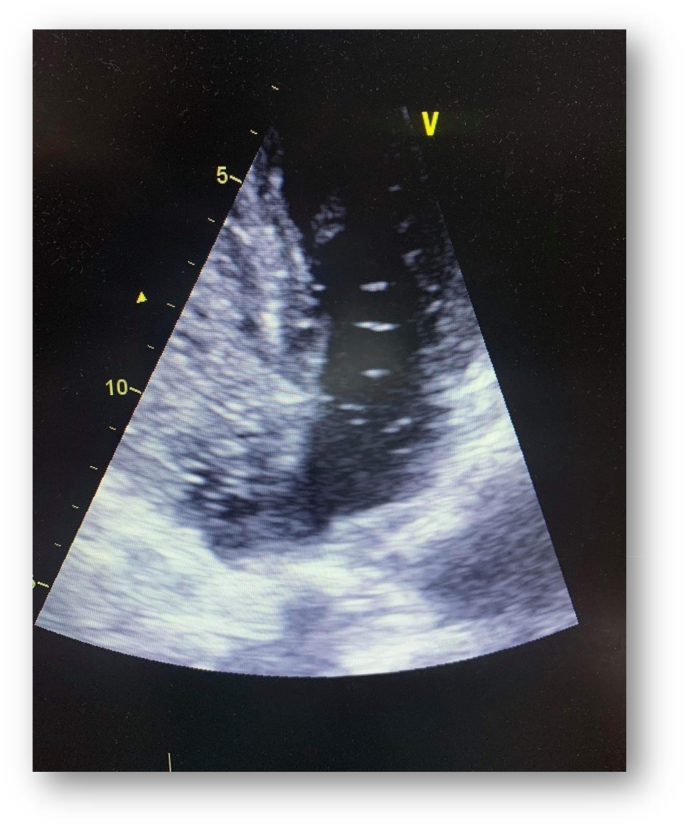
the apparence of more than 3 bubbles of the left side of the heart at the TTE with bubble test.

At the etiological assessment: Thrombophilia tests were without abnormalities, and since the patient did not tolerate the transesophageal echocardiography (TOE), we realized a TTE with bubble test confirming then the diagnostic of PFO by the appearance of more than 3 bubbles on the left side of the heart after the injection of sterile saline into a peripheral vein, during the first three beats, with the Valsalva maneuver (Fig. 5).

A treatment based on anticoagulant and platelet antiaggregant were prescribed on discharge of the patient, with regular follow up at the consultation.

## 3 Discussion

The association of mesenteric involvement and acute ischemia of the upper extremity is extremely rare. In our case, it was observed as part of paradoxical embolism associated with a patent foramen oval (PFO).

Indeed, The oval foramen (called also ostium secundum of Born) is an interatrial congenital anatomical communication, allowing during the intra-uterine life, the passage of oxygenated blood from the placenta; from the right atrium to the left atrium; thus towards the large systemic circulation. After birth the pressure drop in the right cavities; which becomes lower than that in the left cavities and then causes the closure of the septal valve of the foramen [10].

The paragraph of autopsy case study is elmimnated due to its little value.

A pressure gradient is maintained between the right and left cavities under physiological conditions causing the passive closing of the PFO. However, sometimes the pressure in the right atrium can exceed the one in the left atrium. Allowing the passage of particulate such a thrombi into the systemic circulation. It is the case for instance during the Valsalva manoeuvres ([11,12]) or when a patient suffers pulmonary embolism [14]. Which is the case of 85% cases of paradoxical embolism [6] or other causes of pulmonary arterial hypertension; right atrial hyper pressure by tricuspid valve disease [12], right ventricular hyper pressure, right ventricular failure [12], positive pressure ventilation, positive end-expiratory pressure ventilation, right ventricular infarction, hypoxemia, extracorporeal circulation, pneumonectomy, chronic liver diseases, gas embolism [13].

FOP is frequently associated with various anatomical variations such as the Eustachian valve [15], Chiari malformation [16] or the aneurysm of the interatrial septum (ASA). The ASA is in 2% of clinical studies [17]. It is characterized by a 10 mm septal excursion with a base diameter of 15 mm. Most of the patients with a PFO indeed have an ASA since the ASA generates the PFO [7]. Patients with ASA have an increased risk of paradoxical embolism than patients not suffering from it. In addition, the annual risk of recurrence increases from one to 4% per year when a PFO is associated with an ASIA [18].

PFO is most often asymptomatic but it can cause manifestations with variable degrees of severity. Among these manifestations, there is the paradoxical embolism that is due to the migration of thromboembolic particles from the venous circulation to the systemic circulation through the patent foramen. The areas of paradoxical embolism are represented in decreasing order of frequency by the peripheral (49%), cerebral (37%), coronary (9%), renal (1%) and spleen arteries (1%) [6]. The mesenteric involvement similar to the one described in our patient is particularly rare.

Paradoxical embolism reaches several areas as described in the literature; This is the case of our patient who presented mesenteric ischemia with acute ischemia of the right upper limb.

According to Meacham et al.; out of three patients (27%) with paradoxical arterial embolism were affected in different areas: one with cerebral and peripheral involvement, one with splenic, mesenteric and peripheral involvement and one with carotid, renal, splenic, mesenteric and peripheral involvement [19].

Several studies highlighted the solid association between a PFO and a cryptogenic stroke with a relative risk of 6.0 (95% CI: 3.7 to 9.7) for patients suffering from a PFO and a cryptogenic stroke compared to those whose cause of the stroke has been identified [20].

When the paradoxical embolism causes a myocardial infarction the prognosis is generally fatal. However, this phenomenon remains poorly understood in the literature.

PFO can be detected by Echographic techniques: transthoracic echocardiography (TTE), transesophageal echocardiography (TEE), or transcranial echocardiography (TCE).

TTE is a simple, non-invasive investigation that is always available. As color Doppler detects only 5%–10% of inter-atrial shunts (28 art) patients with suspected PFO should undergo a bubble test. But the poor sensitivity compared to the TEE is the principal limitation of the TTE. So a TEE with color Doppler should complete every negative transthoracic study, it's considered as the most reliable diagnostic test [21].

In our case, since the patient did not tolerate the TEE, which is a absolute contraindication of this echocardiographic technique, we realized a TTE with bubble test confirming then the diagnostic of PFO.

Paradoxical embolus requires in most of the cases an operative intervention: Critical limb ischemia due to paradoxical embolism involve an urgent embolectomy, the ischemia of the mesenteric circulation is, also considered as an emergency requiring to be treated by embolectomy.

In terms of medical therapy, patients with paradoxical embolism are universally treated with long-term anticoagulation therapy and evaluated for PFO repair. In case of contraindication of the anticoagulation an IVC can be proposed to prevent further pulmonary emboli, but it may not trap small emboli.

Moreover, all treated patients should be evaluated for elective PFO closure ([22,23]). but in our case, we didn't recommended the closure of the PFO because of the small size of the shunt and specially because the patient refuses that procedure. And since there is no contraindication of the anticoagulation in our case, we preferred long time anticoagulation with Acenocoumarol (because of low socio-economic level of our patient).

## 4 Conclusion

Due to its rare frequency, paradoxical embolism remains a pathology rarely mentioned by clinicians, although it can engage the functional and vital prognosis of the patient, hence the interest of a good cardiac evaluation in any patient with embolic ischemia. The management of this patholology is multidisciplinary involving surgical and long term medical treatment.

## Ethical approval

Applicable.

## Sources of funding

None.

## Author's contribution

Y. Banana: conception, literature review, analysis, data collection, writing- review & editing.

A. Rezziki: conception, analysis, data collection, writing- review & editing.

O. Kallel: conception, software, writing- review & editing.

H. Rasras: conception, software, writing- review & editing.

N. El ouafi: conception, methodology, supervision.

Z. Bazid: conception, methodology, supervision.

O. El mahi: conception, methodology, supervision.

A. Benzirar: conception, methodology, supervision.

## Registration of research studies

Name of the registry:

Unique Identifying number or registration ID:

Hyperlink to your specific registration (must be publicly accessible and will be checked).

## Guarantor

Pr. Noha El ouafi: el.nouha@hotmail.com.

(Department of Cardiology & Laboratory of Epidemiology, Clinical Research and Public Health.

Mohammed VI University Hospital of Oujda, Mohammed First University of Oujda, Morocco).

Pr. Zakariae Bazid: bazidzakaria@gmail.com.

(Department of Cardiology & Laboratory of Epidemiology, Clinical Research and Public Health.

Mohammed VI University Hospital of Oujda, Mohammed First University of Oujda, Morocco).

Pr. Omar El Mahi: omarelmahi@yahoo.fr.

(Department of Vascular Surgery & Laboratory of Epidemiology, Clinical Research and Public Health.

Mohammed VI University Hospital of Oujda, Mohammed First University of Oujda, Morocco).

Pr. Adnane Benzirar: b.adnane@yahoo.fr.

(Department of Vascular Surgery & Laboratory of Epidemiology, Clinical Research and Public Health.

Mohammed VI University Hospital of Oujda, Mohammed First University of Oujda, Morocco).

## Source of financial support

There's no financial support

## Consent

The patient is consent for publication.

## Provenance and peer review

Not commissioned, externally peer-reviewed.

## Declaration of competing interest

Non-conflicts.
